# Imaging in Third Molar Surgery: A Clinical Update

**DOI:** 10.3390/jcm12247688

**Published:** 2023-12-14

**Authors:** Adib Al-Haj Husain, Bernd Stadlinger, Sebastian Winklhofer, Fabienne A. Bosshard, Valérie Schmidt, Silvio Valdec

**Affiliations:** 1 Clinic of Cranio-Maxillofacial and Oral Surgery, Center of Dental Medicine, University of Zurich, 8032 Zurich, Switzerland; adib.al-hajhusain@zzm.uzh.ch (A.A.-H.H.); bernd.stadlinger@zzm.uzh.ch (B.S.); fabienne.bosshard@zzm.uzh.ch (F.A.B.); valerie.schmidt@zzm.uzh.ch (V.S.); 2 Department of Neuroradiology, Clinical Neuroscience Center, University Hospital Zurich, University of Zurich, 8091 Zurich, Switzerland; 3 Department of Radiology, Hirslanden Zurich, 8032 Zurich, Switzerland; sebastian.winklhofer@hirslanden.ch

**Keywords:** third molar, wisdom teeth, third molar surgery, dental extraction, oral surgery, oral radiology, panoramic radiography, computed tomography, cone-beam computed tomography, magnetic resonance imaging, photon-counting computed tomography

## Abstract

Third molar surgery is one of the most common surgical procedures performed in oral and maxillofacial surgery. Considering the patient’s young age and the often-elective nature of the procedure, a comprehensive preoperative evaluation of the surgical site, relying heavily on preoperative imaging, is key to providing accurate diagnostic work-up, evidence-based clinical decision making, and, when appropriate, indication-specific surgical planning. Given the rapid developments of dental imaging in the field, the aim of this article is to provide a comprehensive, up-to-date clinical overview of various imaging techniques related to perioperative imaging in third molar surgery, ranging from panoramic radiography to emerging technologies, such as photon-counting computed tomography and magnetic resonance imaging. Each modality’s advantages, limitations, and recent improvements are evaluated, highlighting their role in treatment planning, complication prevention, and postoperative follow-ups. The integration of recent technological advances, including artificial intelligence and machine learning in biomedical imaging, coupled with a thorough preoperative clinical evaluation, marks another step towards personalized dentistry in high-risk third molar surgery. This approach enables minimally invasive surgical approaches while reducing inefficiencies and risks by incorporating additional imaging modality- and patient-specific parameters, potentially facilitating and improving patient management.

## 1. Introduction

Third molar surgery is one of the most frequently performed outpatient procedures in everyday oral and maxillofacial surgery. In most cases, it is a straightforward procedure with minimal risk of permanent damage. The implementation of time-efficient, minimally invasive surgical approaches, along with state-of-the-art techniques such as piezoelectric surgery, laser-assisted surgery, and 3D imaging and navigation into routine workflows, enables personalized patient care in a multidisciplinary, coordinated setting, improving precision, safety, and the patient experience [1,2,3]. To ensure a successful surgical outcome and to address potential complications that may arise from conditions, such as impaction, pericoronitis, and crowding, proper surgical planning by the performing surgeon is of decisive importance. This involves a thorough preoperative understanding of the anatomical complexity and variability of the size and shape of the third molars, their positional relationships to adjacent vulnerable anatomical structures, and concomitant pathologies of the surgical site [4].

When deciding whether to retain or extract a third molar, the risk factors that favor tooth retention must be distinguished from those that make extraction more difficult, always keeping in mind the potential for postoperative complications that may affect the success of the procedure and the patient’s recovery. The assessment of symptomatic patients includes a comprehensive clinical and radiological evaluation, with common indications for surgical removal of the third molar being pain-related complaints, crowding, recurrent swelling, and surgical site infection [5]. Postoperative complications are a potential risk of any surgical procedure. The major complications of third molar surgery are diverse and include a wide range of postoperative challenges that may require early patient-specific management within the appropriate therapeutic time window, followed by a thorough follow-up. In the mandible, transient iatrogenic injuries to the branches of the mandibular nerve, namely the inferior alveolar nerve (0.4–8.4%) [6,7] and the lingual nerve (0.01–2%) [8], although rare, often lead to sensory impairment and limitation of daily activities, significantly affecting patients’ quality of life. In the maxilla, there is also a potential risk of creating an iatrogenic opening in the maxillary sinus during surgery [9]. Other perioperative complications inherent to any dentoalveolar surgical procedure include the possibility of leaving behind tooth fragments, as well as potential complications, such as pain, bleeding, swelling, infection, alveolar osteitis, or temporomandibular joint dysfunction [9]. However, the course of the third molar surgical procedure is influenced by several variables, including the patient’s age, the surgeon’s level of experience, and the tooth’s impaction depth at the surgical site [10,11]. Nonetheless, these risks can be significantly reduced with a careful preoperative assessment and personalized treatment planning. This assessment includes a thorough indication-specific, modality-oriented preoperative radiologic evaluation, patient preparation, a minimally invasive aseptic surgical technique aimed at accurate hard and soft tissue management, and adequate adherence to postoperative instructions [12].

The radiological workflow in the context of third molar surgery involves the use of various imaging modalities to enhance preoperative assessments and refine surgical planning. Notably, conventional X-ray-based two-dimensional orthopantomography (OPG), obtained with a relatively low radiation exposure, is frequently performed as the initial examination prior to third molar surgery and is often sufficient to assess the positional relationships and adjacent anatomical information at the surgical site [13]. However, the limitations of OPGs become evident when dealing with cases characterized by complex anatomical variations or in scenarios where precise three-dimensional information is imperative due to the presence of radiographic risk signs, such as an overlap of anatomical structures or displacement of the mandibular canal [14]. Three-dimensional radiological imaging techniques, such as computed tomography (CT) and cone-beam computed tomography (CBCT), are essential for diagnosis and treatment in terms of topographical information, resolution, and dimensional accuracy in high-risk surgery [14]. Although characterized by standardized grayscale values and limited soft tissue imaging resolution, CBCT has emerged as the gold standard in oral and maxillofacial surgery for the visualization of craniofacial and dental bony structures. Its superiority is emphasized by its greater accessibility, shorter scan times, reduced radiation exposure, and cost-effectiveness compared to the conventional CT scan [4,14]. In the context of third molar surgery, numerous studies have demonstrated that CBCT generates high-resolution cross-sectional images that provide detailed insight into morphological features and irregularities of surgically relevant parameters, such as the mandibular canal’s precise buccolingual position and cortical integrity [15,16]. 

Recent advancements in the field of biomedical imaging, including artificial intelligence and machine learning, are reshaping the landscape of radiological workflows. Among the emerging X-ray-based technologies, photon-counting computed tomography (PCCT) stands out as a promising tool with the ability to redefine established approaches to preoperative assessments and high-risk surgical planning. With its unique ability to quantify individual X-ray photons, PCCT has attracted attention as a potential alternative to CTs or CBCTs [17]. It offers advantages such as faster scanning speeds, enhanced soft tissue contrast, and superior artifact reduction. These features have the potential to substantially increase surgical precision and positively impact patient outcomes by providing a more comprehensive depiction of anatomical structures and positional relationships [18]. Additionally, integrating artificial intelligence (AI) and machine learning (ML) techniques into oral and maxillofacial surgery has provided opportunities to improve preoperative assessments, treatment planning, and postoperative management. However, the widespread use of X-ray-based imaging modalities in oral and maxillofacial practice, particularly three-dimensional imaging with its associated elevated radiation doses [19], could potentially contribute to an increased lifetime susceptibility to radiation-induced malignancies [20]. Thus, the principles of ALARA (As Low As Reasonably Achievable) and ALADA (As Low As Diagnostically Acceptable) have become integral aspects of clinical decision making in the continuing effort to minimize or potentially eliminate radiation exposure from medical imaging [21]. Magnetic resonance imaging (MRI), which provides an accurate and viable radiation-free alternative to CBCT for highly detailed imaging of the third molar region, has made significant advances through a variety of technical improvements and the development of novel dental MRI-specific sequences [22,23]. By providing a comprehensive visualization of soft tissue structures, anatomical relationships, and potential pathologies, MRI is emerging as a valuable tool for enhancing preoperative assessments, planning, and decision making in complex third molar surgical cases.

Considering the heterogeneity of data in the literature and the use of many available imaging modalities prior to third molar surgery, the aim of this article is to provide an up-to-date overview of the use of all available imaging techniques. To this end, the existing literature is comprehensively reviewed and critically evaluated, highlighting the potential indication-specific, modality-oriented benefits and limitations based on current scientific evidence and clinical experience.

## 2. Imaging Modalities in Third Molar Surgery 

### 2.1. Orthopantomography (OPG)

**Figure 1:**
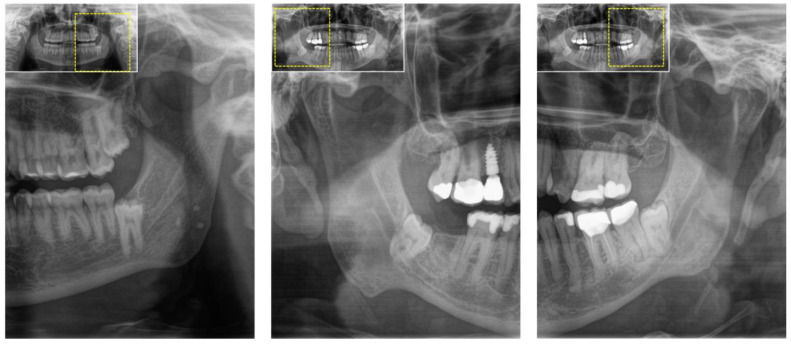
Two-dimensional orthopantomography (OPG) was initially performed in patients with a clinical indication for third molar surgery. This radiographic assessment reveals several radiographic risk signs, including the overlap of the third molar and mandibular canal, as well as incomplete of the osseous boundaries of mandibular canal. In such high-risk cases, three-dimensional imaging, such as cone-beam computed tomography (CBCT), is recommended to improve preoperative assessments and refine surgical planning.

In most cases, an OPG, a widely used extraoral two-dimensional dental radiograph, is routinely obtained during the initial examination of a symptomatic patient prior to third molar surgery. It provides a comprehensive panoramic view of the oral cavity and adjacent structures with a relatively short exposure time and dose (4–30 μSv) [24]. It is considered a valuable tool for assessing several critical factors relevant to the surgical site, such as root number, shape, curvature, root resorption, positional relationships, angulation to adjacent teeth, proximity to vital structures such as the mandibular canal or maxillary sinus, and the presence of additional pathology [25]. Its main advantage is the low radiation exposure to the patient, coupled with the ability to acquire information on all four third molars from a single radiograph. OPGs reliably depict vertical positioning and mesiodistal angulation, but the limitations of OPGs as a stand-alone technique become apparent when assessing the bucco-lingual spatial relationships. In addition, the technique’s inherent distortion and magnification effects can lead to inaccuracies in assessing the precise relationship between the impacted tooth and surrounding anatomical structures [4], particularly in the interpretation of cases involving overlapping molars, where the contact or non-contact position with the adjacent teeth cannot be accurately distinguished because the two-dimensional radiographic representation differs from the actual clinical position. Thus, despite their advantages, OPGs have limitations that can affect the understanding of the potential risks and complications that every surgeon needs to be aware of. Specifically, for the preoperative localization of the mandibular canal and its relationship to the third molar, OPGs only provide information about the canal in the horizontal plane. The presence of radiographic risk signs, such as a close overlap of the third molar and the mandibular canal, diversion of the mandibular canal, darkening of the roots, and incomplete integrity of the osseous margins of the mandibular canal, can increase the risk of inferior alveolar nerve injury up to threefold [16], necessitating preoperative three-dimensional imaging modalities such as CT or CBCT [14,26] (Figure 1). Information regarding the integrity of the cortical boundaries of the mandibular canal is of particular importance to the performing surgeon, as it influences the surgical approach, especially in the context of a vestibular surgical approach utilizing a mucoperiosteal flap with an intrasulcular bayonet incision, and assesses the risk of a possible dislocation of tooth fragments towards the floor of the mouth and sublingual space. In the maxilla, complex root configurations are less problematic due to the presence of cancellous bone. Extreme palatal positions are rare and can be quickly identified intraoperatively, allowing the surgical approach to be quickly modified without significantly increasing morbidity [27]. While the positional relationship of the third molar roots to the maxillary sinus is of clinical importance, the occurrence of an inadvertent oroantral fistula can be managed without major complications in most cases with an effective surgical technique. However, retromaxillary, pterygopalatal, and infratemporal dislocations of the third molar are of greater concern [27]. Despite these concerns, three-dimensional imaging does not significantly increase the value of risk assessments in these cases. However, in cases involving supernumerary third molars, three-dimensional imaging surpasses conventional OPGs in its diagnostic superiority [28]. Postoperatively, an OPG may be performed to confirm the successful removal of the third molar and to assess any changes in the adjacent structures or to perform periodic long-term monitoring in cases with complications or at risk for late-onset alterations, such as cystic changes, to monitor the patient’s current condition, rule out root remnants or further complications, and ensure proper healing. In summary, OPG is critical in evaluating and planning of third molar surgery. It serves as a first step in assessing the pathoanatomy of the surgical site and helps identify potential perioperative risks. However, its limitations must be carefully considered, and in cases that require a more comprehensive understanding of the anatomical intricacies, the integration of three-dimensional imaging techniques, such as CBCT, becomes essential to improve surgical precision and patient safety.

### 2.2. Computed Tomography (CT) 

Conventional CT uses complex multidirectional motion to acquire high-quality three-dimensional cross-sectional images of the dentomaxillofacial region of interest. Although spiral CT or multidetector CT (MDCT) is rarely used for preoperative evaluation in third molar surgery, mainly due to limited availability in the dental office, increased radiation exposure, potential need for contrast agents, and increased cost, it plays an essential role in oral and maxillofacial surgery [29]. 

**Figure 2:**
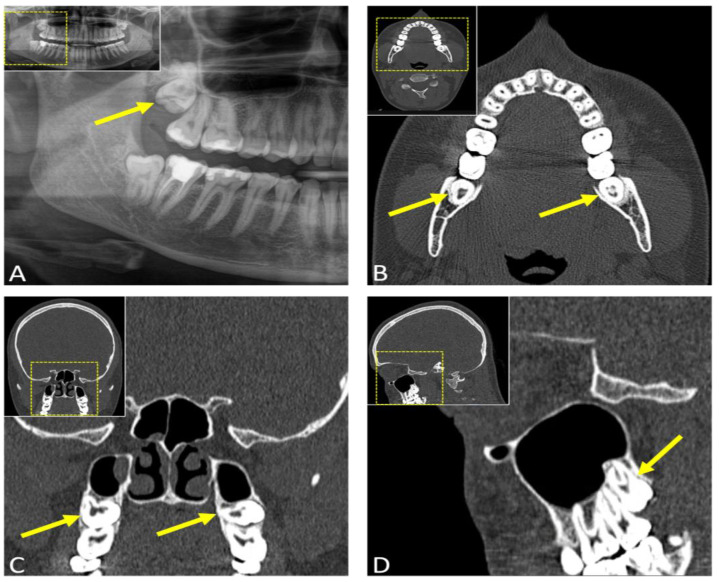
(**A**) Panoramic radiography (OPG) and (**B**–**D**) computed tomography (CT) of a 29-year-old female patient with an indication for third molar surgery. (**A**) The arrow points to the third molar in the upper first quadrant, while (**B**) axial, (**C**) coronal, and (**D**) sagittal CT reconstructions depict the upper third molars.

CT, which provides volumetric imaging and generates multiple high-quality reconstructions in different planes, is particularly advantageous for routine tumor diagnosis and computer-assisted surgical procedures. CT-based surgical planning allows the surgeon to tailor the surgical approach to the patient’s specific anatomy, facilitating the surgical procedure, and enabling the identification of perioperative complications. Studies on the clinical relevance of preoperative CT in third molar surgery have demonstrated its feasibility and accuracy, despite limited soft tissue resolution, improving the surgeon’s understanding of anatomical relationships and preoperative identification of potential complications [4,30]. CT images provided a precise representation of the position of the inferior alveolar canal, its cortication status, and an assessment of its positional relationship to the mandibular third molar [29]. CT scans of cases where the inferior alveolar canal is located lingually to the third molar suggest an elevated risk of compression during extraction, with reports estimating the predictive value of CT for assessing IAN damage to be approximately 20–30% [30,31]. Furthermore, the estimated dimensions of the cortical defect, determined from CT scans by identifying interruptions in the hyperdense cortical boundaries of the inferior alveolar canal, demonstrated a strong correlation with inferior alveolar nerve injury [30] (Figure 2). 

However, it is important to recognize the potential drawbacks associated with CT scans, particularly the increased radiation exposure. While it has long been considered a high-dose technique, the development of MDCT and low-dose protocols tailored for indication-specific modality-oriented imaging has rendered this perception obsolete, as doses below 0.15 mSv can now be achieved [32,33]. In summary, the decision to use CT depends on the complexity of the case, considering additional imaging modalities and patient-specific parameters, such as additional pathologies, radiation exposure considerations, and equipment availability, to ensure that the benefits outweigh the potential risks. However, in routine clinical use for third molar surgery requiring three-dimensional imaging, CBCT has predominantly replaced CT scans. Today, the use of CT in the perioperative setting is limited to complex, high-risk surgical procedures that require highly detailed, three-dimensional anatomical information to address intricate surgical challenges. 

### 2.3. Cone-Beam Computed Tomography (CBCT) 

Similar to conventional CT, CBCT falls within the category of X-ray techniques that use mathematical algorithms to generate a reconstructed image from a multitude of individual X-ray images. The primary distinction between CT and CBCT lies in the beam geometry employed and, consequently, the type of digital detector used. While conventional CT uses a fan-shaped beam coupled to one or more one-dimensional rows of detectors, CBCT was originally developed with a cone-shaped beam detected by a two-dimensional detector. However, modern CBCT scanners have evolved beyond the use of cone-shaped beams. Instead, pyramid-shaped beams and flat panel detectors are used for three-dimensional image reconstruction, enabling the acquisition of essential data relevant to volume reconstruction within a single rotation [34,35]. In clinical practice, this results in images with superior spatial resolution compared to conventional CT scanners, usually accompanied by lower radiation doses. However, doses reported in the literature range from 0.05 to 0.6 mSv [36]. A number of CBCT systems have been developed specifically for hard tissue imaging in dentomaxillofacial imaging. Despite the inherent limitations of CBCT in terms of soft tissue contrast and standardized grayscale values, the increased use in dentoalveolar surgical procedures can be explained by the greater accessibility, shorter scan times, and cost effectiveness of CBCT compared to conventional CT [37]. 

**Figure 3:**
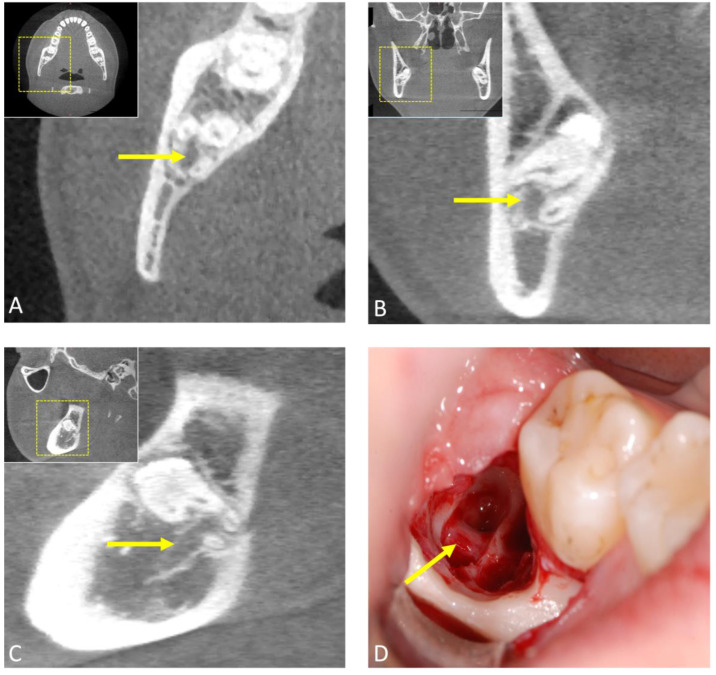
Preoperative cone-beam computed tomography (CBCT) scan of a patient undergoing mandibular third molar surgery showing radiographic risk signs in two-dimensional X-ray based orthopantomography. (**A**) Axial, (**B**) coronal, and (**C**) sagittal image reconstructions showing the high-risk relationship between the mandibular third molar and the mandibular canal. The arrow indicates the inferior alveolar canal. Image (**D**) shows the intraoperative situation after surgical removal of the third molar and the precise anatomical localization of the inferior alveolar nerve (arrow).

**Figure 4:**
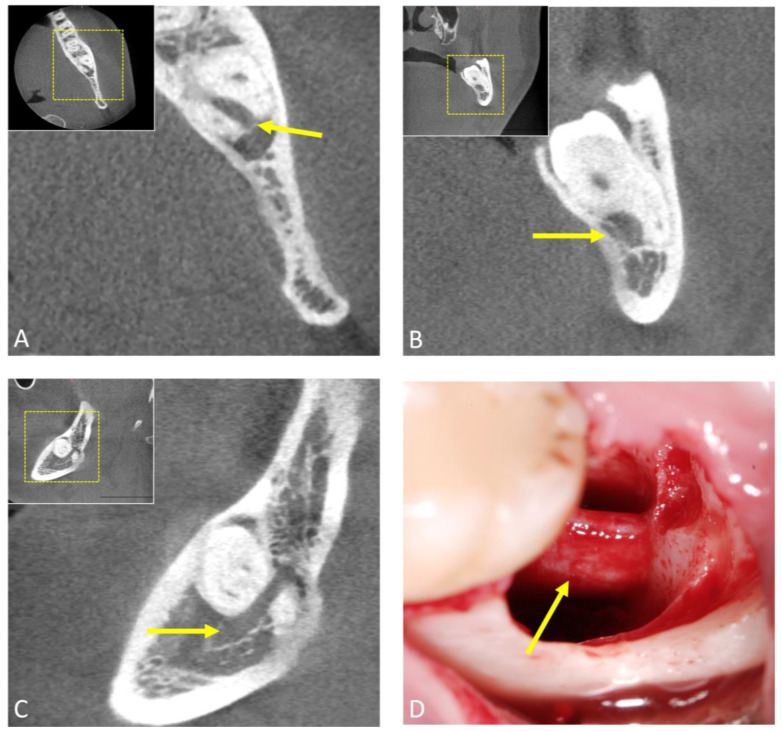
Preoperative cone-beam computed tomography (CBCT) scan of a patient undergoing mandibular third molar surgery. (**A**) Axial, (**B**) coronal, and (**C**) sagittal image reconstructions showing the preoperative high-risk anatomical relationships. The arrow indicates the inferior alveolar canal. Image (**D**) shows the intraoperative situation after surgical removal of the third molar and the precise anatomical location of the inferior alveolar nerve (arrow).

The application of CBCT in third molar surgery has reshaped the landscape of radiological workflows, providing comprehensive three-dimensional insights that significantly enhance preoperative assessments and surgical planning. With its sub-millimeter isotropic spatial resolution, this advanced imaging modality is increasingly complementing or replacing conventional radiological techniques in routine clinical practice for indicated cases by offering arbitrary reconstructions that visualize anatomical complexities in the third molar region without superimposition. Numerous studies have confirmed the feasibility and accuracy of CBCT in the radiological workflow for third molar surgery [38]. These images are obtained with low radiation exposure, typically in the range of approximately 18 to 200 μS [39]. In particular, CBCT excels at visualizing surgically relevant morphological features and positional anomalies, especially the mandibular canal’s precise buccolingual position and cortical integrity, thus assessing the risk of nerve injury. In this regard, it significantly surpasses two-dimensional OPG’s capabilities in assessing perioperative risks [16,40]; however, data from the literature suggest that the use of CBCT does not reduce neurosensory dysfunction compared to OPG [41]. While various studies have attempted to correlate clinical and radiological factors with surgical difficulty, the proposed indices are infrequently used as they have been shown not to consistently align with actual surgical challenges [42,43,44]. Recent evidence suggests that both clinical and radiographic variables in combination are critical in predicting surgical difficulty in impacted third molar surgery [45]. However, in indicated cases, in impacted and displaced third molars or when the inferior alveolar nerve is located lingually, 3D diagnostics may lead to a modified surgical approach [46], such as coronectomy in certain complicated cases [47], resulting in a lower rate of transient nerve damage [48]. As with intra-operative imaging, CBCT may be necessary in complex cases or when complications arise during surgery to offer real-time guidance to the surgeon. In some instances, it can also aid in post-operative follow-ups (Figure 3 and Figure 4).

The utilization of dedicated low-dose CBCT protocols in oral and maxillofacial surgery has gained considerable attention in recent years due to its potential benefits in patient management [49]. Low-dose protocols offer significantly lower radiation exposure and thus increased safety, especially in genetically susceptible adolescents, as well as increased patient comfort through shorter examination times [20,50]. However, limited accessibility, lack of validation, and compromised image quality are limitations that should be weighed against radiation dose and image quality according to the clinical need. It is important to note that although low-dose CBCT offers significant advantages in terms of radiation safety, it is not completely free of radiation exposure and should, therefore, be used with caution. Overall, low-dose protocols that offer confidential diagnostic assessments while maintaining an improved benefit/risk ratio per the ALADA principle show promise as a potential primary diagnostic tool, especially for radiological follow-ups in the management of dentoalveolar surgical procedures. 

The limitations of CBCT become evident in specific complex medical conditions, indicating the need for CT when there is a suspicion of soft tissue involvement by bone tumors or extensive fractures potentially affecting the skull. In cases involving soft tissue tumors, functional temporomandibular joint symptoms, or the need for direct visualization of neural tissue, MRI emerges as the preferred imaging modality, surpassing both CBCT and CT. 

### 2.4. Photon-Counting Computed Tomography (PCCT)

In recent years, there has been considerable progress advancements in optimizing existing CT technology, resulting in faster image acquisition, improved temporal resolution, reduced radiation exposure, and lower contrast agent volumes. However, the detector side of CT technology has seen comparatively less development. A breakthrough is now emerging with the introduction of photon-counting CT scanners, which introduce a completely new detector design to clinical radiology, resulting in ultra-high-resolution images in dental imaging [51]. Thus, among the emerging X-ray-based technologies, photon-counting computed tomography (PCCT) stands out as a promising tool with the ability to redefine established approaches to preoperative assessments and high-risk surgical planning. 

Due to its unique ability to quantify individual X-ray photons, PCCT has attracted attention as a potential alternative to CT or CBCT [17]. It features benefits such as faster scanning speeds, improved soft tissue contrast, and superior artifact reduction. With a spatial resolution of up to 200 μm, it offers similar resolution to high-resolution CBCT (80–125 μm) [51]. The major technological advancement in the field of PCCT is primarily due to the revolution in X-ray detectors. Whereas conventional CT systems typically use scintillator materials to convert incoming X-ray photons into visible light before decoding them, PCCT instead uses sensors consisting of a single layer of semiconductor diodes. This innovative approach allows each absorbed X-ray photon to create a unique charge cloud. These charge clouds are then individually transported to the detector pixels by applying a bias voltage, bypassing the initial step of converting X-rays to visible light, which is common in CT detectors. In addition, the absence of a scintillator layer and reflective lamellae in PCCT detectors contributes to a smaller pixel size compared to their conventional CT counterparts, significantly increasing image resolution [51,52,53]. 

In the context of third molar surgery, this advancement can offer unprecedented levels of anatomical detail, potentially substantially increasing surgical precision and positively impacting patient outcomes by providing a more comprehensive depiction of anatomical structures and positional relationships at the surgical site [18]. However, the utilization of this promising imaging technology in dentistry is still in its infancy, with comprehensive evidence and comparative studies of its feasibility and efficacy in third molar surgery particularly lacking. As PCCT technology continues to develop and takes its first steps towards integration into clinical practice, this should always be considered when interpreting the current state of research in this field.

### 2.5. Magnetic Resonance Imaging (MRI) 

MRI, with its exceptional soft tissue contrast and non-invasive nature, has recently been proposed as an accurate and reliable radiation-free 3D imaging modality for dentoalveolar imaging. It offers the potential to provide highly detailed images of the third molar region, presenting a viable alternative to, and in some cases, a potential replacement for CBCT, as indicated in [23]. However, this transition to a radiation-free alternative, which is in line with the ALADA principle’s emphasis on minimizing or possibly eliminating radiation exposure, presents several challenges, given the heavy reliance on X-ray-based imaging in the dental field. 

**Figure 5:**
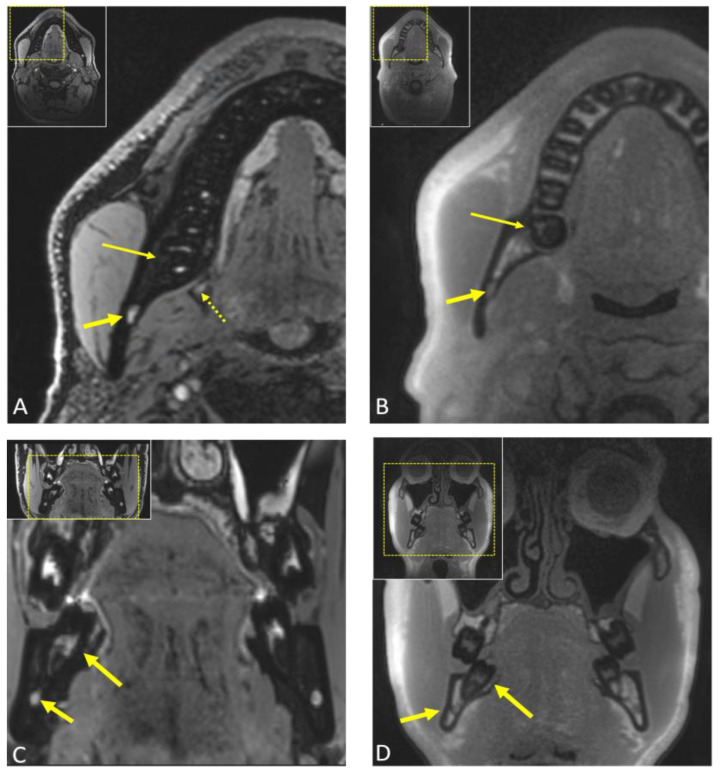
(**A**) Axial and (**C**) coronal reconstruction of a preoperative 3D double-echo steady-state (DESS) MRI of a patient undergoing third molar surgery [56]. In (**A**), the long arrow indicates the mandibular third molar, while the short arrow points to the entry of the inferior alveolar nerve, depicted as a hyperintense signal in the mandibular canal. The dotted arrow points to the lingual nerve at the level of the third molar. In (**C**), the long arrow points to the tooth 48, and the short arrow visualizes the precise localization of the inferior alveolar nerve within the osseous boundaries of the mandibular canal. (**B**) Axial and (**D**) coronal images display reconstructions from a preoperative 3D ultrashort echo time (UTE) MRI, which is an MRI sequence known for producing CT-like MRI images, particularly suitable for imaging osseous tissue. In (**B**,**D**), the long arrow points to the mandibular third molar, while the short arrow points to the inferior alveolar nerve.

MRI has been used in dental radiology for more than three decades for various indications, allowing the visualization of anatomical and pathological conditions in the oral cavity. Its distinctive capability to simultaneously visualize soft and hard tissues within the dentoalveolar complex without radiation exposure has established it as a valuable tool for the early detection and visualization of oral and maxillofacial diseases [54]. Existing studies confirm the feasibility and accuracy of preoperative MRI in visualizing the third molar region, focusing on critical structures such as the inferior alveolar and lingual nerve [23]. The initial MRI studies used specific imaging protocols, such as the Phase Encode Time Reduction Acquisition (PETRA) MRI sequence, to evaluate lingual nerve visualization and various relevant quantitative and qualitative parameters associated with third molar surgery [55]. These older MRI studies employed low-magnetic-field strengths and conventional MR sequences, including T1-weighted gradient-echo (GE) and fast spin-echo (SE) sequences. This resulted in extended scan times, suboptimal image quality with a lower signal-to-noise ratio, and inadequate resolution with slice thicknesses of up to 4 mm, making them unsuitable for routine clinical applications. However, it was possible to accurately depict the inferior alveolar neurovascular bundle as a hyperintense signal with good contrast, primarily because of the lower signal from the surrounding bony margins of the mandibular canal. In recent years, dental-dedicated MRI has made significant progress through a number of technical improvements and the development of novel dental MRI-specific sequences [22,23]. Recent advances in MRI protocols for dental imaging have introduced specialized “black bone” MRI sequences, such as the 3D double-echo steady-state (DESS) and 3D short tau inversion recovery (STIR) sequences. These sequences provide high-resolution, high-contrast images, enabling simultaneous visualization of the inferior alveolar nerve within the mandibular canal’s osseous boundaries [56,57]. In these dedicated MRI protocols, which employ water excitation/fat suppression techniques, the nerve tissue exhibits distinct hyperintense signals and can be easily differentiated from adjacent osseous structures, such as the mandibular canal, due to the presence of the myelin sheath surrounding the nerves [58]. This represents a significant advantage in terms of the preoperative radiological workflow in third molar surgery, particularly in the direct focal and continuous visualization of nerve tissues, such as the lingual nerve [59] and the inferior alveolar nerve [56] (Figure 5). 

**Figure 6:**
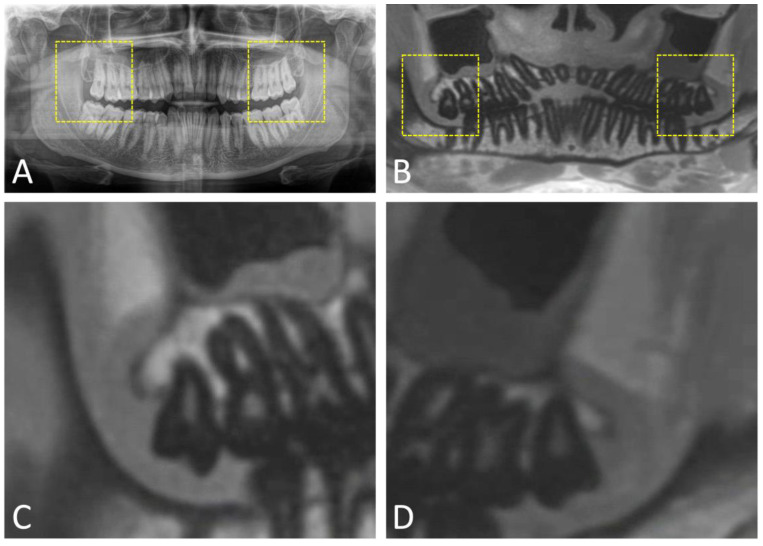
(**A**) X-ray-based orthopantomography (OPG) and (**B**) overview image of an OPG-like MRI reconstruction (MR-OPG) derived from a 3D ultrashort echo time (UTE) dataset of a 29-year-old male patient. Images (**C**,**D**) illustrate the surgical site in the third molar region, displaying the maxillary third molar in the first and second quadrant.

Furthermore, dental MRI-specific protocols enable a reliable assessment of the spatial relationship of mandibular third molars to anatomical structures relevant to third molar surgery [59,60,61]. These studies have investigated different MRI protocols, each with their own strengths and weaknesses. However, they have been shown to be effective in assessing the relative position of mandibular third molars in relation to the inferior alveolar nerve and to provide significantly better visualization of the mandibular canal than CBCT [23]. Further advancements utilizing ultrashort echo time (UTE) sequences have successfully addressed the limitations of MRI in visualizing bony structures, yielding promising results. This is achieved through ultrashort hard pulse excitation and three-dimensional center-out radial sampling of k space, which produces image quality comparable to standard pulse sequences [62], resulting in CT-like MRI images of the dentomaxillofacial complex [63]. The application of image post-processing techniques has enabled the creation of MR-OPGs in the fields of operative dentistry and oral and maxillofacial surgery. This marks a promising advancement in the diagnostic imaging for these specialties, delivering a comprehensive diagnostic assessment that surpasses the capabilities of conventional OPGs [64] (Figure 6). 

**Figure 7:**
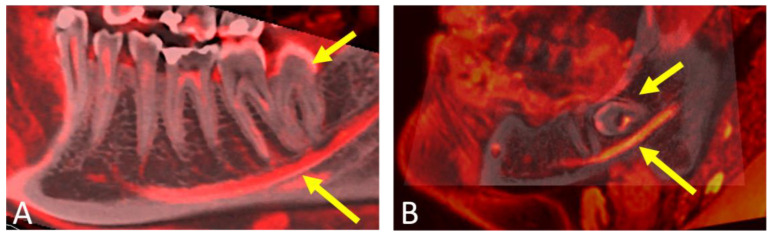
Two sagittal reconstructions—(**A**) for a 32-year-old male patient, and (**B**) for a 19-year-old male patient—derived from a fused dataset of cone-beam computed tomography (CBCT) and magnetic resonance imaging (MRI). In the images, the MRI scan is highlighted in red, while the conventional grayscale values represent the CBCT scan. Short arrows point to the (**A**) distally angulated and (**B**) mesially angulated impacted mandibular third molar, while long arrows point to the course of the inferior alveolar nerve within the osseous boundaries of the mandibular canal relative to the third molar.

Compared to CBCT, DESS and STIR MRI allowed for a practical and highly confidential preoperative assessment without significant limitations in diagnostic information, irrespective of the examiner’s experience [60,61]. In cases with borderline diagnostic criteria, the fusion of CBCT and MRI images into a single dataset may offer distinct advantages over the reliance on conventional CBCT or MRI imaging alone for standard preoperative radiological assessments, especially in the presence of associated pathological conditions [60] (Figure 7). In a separate study, DESS MRI was used to accurately determine the intraosseous location of the inferior alveolar nerve within the mandibular canal. This study showed that the retention type of the third molar exerts an influence on its intraosseous positioning. Specifically, this study observed that the third molar has the potential to displace the alveolar nerve in segments proximal to the contact site. This finding may have significant advantages in preoperative assessments, particularly in complex surgical procedures performed in close proximity to the inferior alveolar nerve [57]. 

Nevertheless, MRI in the oral cavity continues to face certain limitations, including susceptibility to motion artifacts, intricate anatomical pathways involving small blood vessels and nerves, and image distortion stemming from motion artifacts and field inhomogeneities induced by metallic dental restorations [65]. The incorporation of innovations such as intraoral coils [66], radiofrequency coils [67], or mandibular coils [68] into radiological workflows using dedicated dental MRI protocols has effectively minimized the impact of these factors. At the same time, the quality of the imaging of dentomaxillofacial structures has been significantly improved, while the acquisition time has been reduced.

In summary, the future clinical application of dental MRI faces challenges due to its limited availability and high cost. However, as radiation-free MRI undergoes further validation through clinical comparative and cohort studies, it has the potential to mark a significant advancement in the field of personalized dentistry. This advanced imaging technology could revolutionize decision making by providing a comprehensive view of soft tissue structures, anatomical relationships, and potential pathology. This could potentially usher in a new era of advanced perioperative diagnostics, leading to greater precision and safety in third molar surgery.

## 3. Artificial Intelligence and Machine Learning in Third Molar Surgery 

In recent years, the integration of artificial intelligence (AI) and machine learning (ML) techniques into oral and maxillofacial surgery has provided opportunities to improve preoperative assessments, treatment planning, and postoperative management. Several studies have explored the clinical applications of AI models in third molar surgery, developing algorithms to enhance different management stages. One notable application is the assessment of the positional relationship between the third molar and the inferior alveolar nerve in OPGs. This is particularly valuable as determining the exact relationship can be difficult and unreliable, as previous studies have shown [69]. These studies aimed to determine whether there was a contact or non-contact situation between the third molar and the inferior alveolar nerve and whether the inferior alveolar nerve was lingual or buccal to the third molar when they overlapped. The comparison included assessing the performance of AI models against oral and maxillofacial surgeons, experienced radiologists, and inexperienced students. It also evaluated the potential use of different deep convolutional neural networks (CNNs) against each other [70,71,72,73,74]. Model performance showed a higher accuracy in determining the bucco-lingual position compared to the true contact position, with AI outperforming oral and maxillofacial specialists (OMFSs). OMFSs achieved between 53% and 70% accuracy for the true contact position, while AI achieved 72%. For the bucco-lingual position, OMFSs achieved between 32% and 48% accuracy, while AI achieved 81% [70]. Other studies have supported and confirmed these findings, suggesting that AI systems can assist clinicians in making accurate positional judgments [72,73,74,75]. This advantage is particularly significant when the observer lacks experience [71]. A predictive model to assess the eruption potential of third molars using an automated angulation measurement in OPGs may help to determine the surgical indication for extraction, thereby addressing potential controversies [76]. Moreover, AI algorithms can assist by predicting surgical difficulty on OPGs, potentially reducing the risk of postoperative complications [77]. ML algorithms can monitor patients’ postoperative progress by analyzing clinical data, tracking healing, and identifying early signs of complications. In this context, training an artificial neural network with 15 clinical parameters generated a predictive score for postoperative edema [78]. All these aspects can assist surgeons in risk stratification and the development of personalized treatment plans. For more complex surgical procedures, AI can provide real-time guidance, with robotics and augmented reality systems assisting surgeons in navigating complex anatomy and ensuring minimal damage to the surrounding anatomical structures [79].

In summary, the incorporation of AI and ML technologies into third molar surgery promises to increase precision, reduce complications, and ultimately improve patient management. As computational speed continues to increase, leading to exponential data production, and as patient-specific data collection methods evolve, it will become increasingly important for clinicians, data scientists, and ethicists to work together. This collaboration is essential to unlock the full potential of these advances but also to maintain high standards of patient care and ethical conduct. In the near future, AI and ML are likely to become indispensable instruments in the toolkit of oral and maxillofacial surgeons, similar to the broader health sector. Thanks to their learning, recognition, and predicting capabilities, neural network assessments have the potential to assist clinicians in decision making, always bearing in mind that rigorous evaluations by experienced clinicians are and will be required. 

## 4. Clinical Recommendations 

From a clinical perspective, alongside a thorough clinical assessment, the radiological workflow for third molar surgery incorporates multiple imaging modalities to facilitate indication-specific, accurate preoperative assessments and surgical planning. Conventional X-ray-based two-dimensional OPGs, obtained with relatively low radiation exposure, are frequently performed as the initial examination prior to third molar surgery and are often sufficient to assess the positional relationships and adjacent anatomical information at the surgical site. In the cases where OPGs do not accurately depict a close overlap between the third molar and the mandibular canal, or if additional radiographic risk signs are present, such as root darkening and incomplete integrity of the osseous margins of the mandibular canal, the use of three-dimensional cross-sectional imaging, such as CBCT, is recommended. CBCT provides a more detailed and comprehensive visualization of the third molar region, allowing for a thorough assessment of the anatomical relationships and potential challenges. It allows for the precise identification of critical structures, including the position of the inferior alveolar canal, variations in tooth anatomy, and potential pathology. However, it is important to note that CBCT is associated with a higher radiation dose compared to conventional OPGs, and its use should be justified based on the clinical need and individual patient factors. When considering the utilization of CBCT, clinicians should carefully weigh the diagnostic benefits against the radiation risk, especially in young patients or those with a lower risk profile. In the event of perioperative complications, additional imaging modalities such as CBCT and MRI can play a crucial role in the diagnostic process and provide valuable information to guide potential pre-interventions. They can help to identify the cause of complications, such as nerve injury, unexpected anatomical variations, or retained tooth fragments. Although less commonly used in perioperative third molar surgery, MRI can be beneficial in assessing soft tissue complications such as nerve injury, infection, or hematoma that cannot be adequately visualized with radiographic imaging modalities. The integration of AI and ML represents emerging tools that will further enhance future radiology workflows and potentially assist clinicians in decision making. However, it is important to note that despite these advances, rigorous evaluations by experienced clinicians remain and will continue to be essential. All of these aspects contribute to providing useful information to the operating surgeon, thereby improving the safety and precision of personalized therapeutic approaches in high-risk third molar surgery. However, the retrospective nature of this review article should always be considered when interpreting the presented data, which were derived from diverse patient populations in various clinical settings. The recognition of these limitations emphasizes the need for further randomized clinical trials to expand and refine our clinical knowledge.

## 5. Conclusions

In conclusion, the clinical approach to third molar surgery requires the indication-specific use of imaging techniques to ensure accurate preoperative assessments and surgical planning. While conventional X-ray OPGs are often sufficient for initial assessments, cases with complex anatomical relationships can benefit from the greater precision of three-dimensional imaging. The integration of advanced imaging modalities, such as CBCT and MRI, in the context of perioperative complications is proving to be invaluable in the diagnosis and management of unforeseen problems. Looking to the future, the integration of AI and ML promises to further enhance radiology workflows, but the experience of the performing surgeon remains essential and will contribute to the overall improvement in the safety and accuracy of surgical procedures. 

## Data Availability

The complete data presented in this study are available upon request from the corresponding author. The data are not publicly available due to privacy restrictions.
